# Cognitive Remediation Therapy for Adolescents with Anorexia Nervosa—Treatment Satisfaction and the Perception of Change

**DOI:** 10.3390/bs7020023

**Published:** 2017-04-18

**Authors:** Camilla Lindvall Dahlgren, Kristin Stedal

**Affiliations:** Regional Department for Eating Disorders, Division of Mental Health and Addiction, Oslo University Hospital, Ullevål HF, Postboks 4950 Nydalen, 0424 Oslo, Norway; post.psykiskhelse@oslo-universitetssykehus.no

**Keywords:** anorexia nervosa, cognitive remediation therapy, eating disorders, treatment satisfaction, neuropsychology, rigidity, cognitive flexibility, central coherence

## Abstract

Cognitive remediation therapy (CRT) has recently been developed for children and adolescents with anorexia nervosa (AN). It focuses on decreasing rigid cognitions and behaviors, as well as increasing central coherence. Overall, CRT has been proven feasible for young individuals with AN, but little is known regarding the specifics of its feasibility, and the perception of change associated with the intervention. Consequently, the aim of the current study was to explore service users’ perspective on CRT with a specific focus on treatment delivery, treatment content, and perceived change. Twenty adolescents (age 13–18) with AN participated in a 10-session course of CRT. A 20-item treatment evaluation questionnaire was administered at the end of treatment, focusing on four aspects of the intervention: (1) general attitudes towards treatment, (2) treatment specifics, (3) the perception of change and (4) the patient-therapist relation. The main findings suggest high levels of treatment satisfaction, but somewhat limited perceptions of change. The current study is one of the most detailed accounts of adolescents’ perspective on CRT published on eating disorders, and highlights several important aspects of the treatment viewed through the eye of the receiver.

## 1. Introduction

Anorexia nervosa (AN) is an eating disorder (ED) characterized by a persistent restriction of energy intake which leads to significant low body weight, an intense fear of gaining weight or becoming fat, a distorted experience of one’s own body and an amplified sense of the body’s influence on self-worth [1]. Individuals with AN often display rigid behaviors and cognitions characterized by preoccupation with details [2]. Such behaviors and cognitions are predominantly linked to core ED symptoms such as food restriction and preoccupancy with weight and shape, but are also commonly seen in the individual’s approach to exercise routines, work or school, or in relation to family and friends [3]. Inflexibility and preoccupation with details are often explained in terms of neuropsychological dysfunction. Studies in adults with AN have yielded consistent evidence of suboptimal executive functioning, especially in cognitive flexibility [4,5], and in information processing which is often characterized by weak central coherence [6]. Cognitive function in children and adolescents with AN has received less attention, and the extant literature report mixed findings with a number of studies failing to find evidence of poor neuropsychological functioning, whereas others have replicated results similar to those reported in adults [7].

Following the introduction of cognitive remediation therapy (CRT) for EDs in 2005 [8], a number of case and feasibility studies have supported its utility in adults with AN [9], and four randomized controlled trials (RCTs) [10,11,12,13] have sought to investigate the effect of the intervention. For individuals with AN, CRT aims to decrease rigidity and increase the balance between local and global information processing [14,15,16]. It is hypothesized that by alleviating some of these cognitive difficulties, ambivalence towards engaging in other psychological interventions might decrease, rendering the individual more open to the prospect of change [8]. In 2010, the original, adult CRT manual [17] was adapted for children and adolescents [18], and although a number of studies have established its feasibility for this younger group [14,19,20,21,22], there are no published RCT. There is also a paucity in studies evaluating the treatment from the receiver’s perspective. This is in contrast with the increased focus on assessment of client satisfaction in ED health services [23,24]. Additionally, it is well known that that treatment dissatisfaction may lead to treatment delay, failure to engage and, ultimately, to treatment withdrawal [25,26]. The aim of this study is to present post-intervention feedback from a feasibility study of CRT for adolescents with AN, and to examine the results through the aspects of treatment satisfaction and perception of change.

## 2. Materials and Methods

### 2.1. Participants

A total of 20 inpatients (N = 10) and outpatients (N = 10) aged 13–18 (mean = 15.9, SD = 1.6) were recruited from Oslo University Hospital HF, Ullevål, and took part in a CRT feasibility trial [14]. All participants were female and in treatment for AN at the time of inclusion. Based on self-reports of binge eating and compensatory behaviors, 18 participants were classified as having a restricting subtype of AN (AN-R). The two remaining participants fitted the description for a binge-purge subtype (AN-BP). Intelligence was assessed using the Wechsler Adult Intelligence Scale—Third Edition (WAIS-III) [27], the Wechsler Abbreviated Scale of Intelligence (WASI) [28] or the Wechsler Intelligence Scale for Children-Third Edition (WISC-III) [29]. All participants scored within the normal range. There were no significant differences between in- and out-patients in any of the baseline assessment variables. Descriptive data at baseline is presented in Table 1.

### 2.2. Procedure

Participants were assessed using an extensive neuropsychological and psychiatric test battery both before and after receiving CRT. The neuropsychological assessment battery included tests focusing primarily on set-shifting and central coherence, whereas the psychiatric assessment included self-reported measures of depression, anxiety and ED psychopathology. A more detailed account of the assessment procedure and content have been presented elsewhere [3,14,33]. After the initial assessment, participants received, on average, 10 once- or twice-weekly individually delivered sessions, and were then re-tested using the same measures as before the treatment started. In addition, participants were asked to fill out a treatment evaluation questionnaire, allowing for exploration of treatment acceptability and the perception of change after treatment. The study was conducted at the Regional Department for Eating Disorders at Oslo University Hospital, Ullevål, HF. All necessary ethical procedures were followed; the data protection service approved the project, and the Regional Committees for Medical and Health Research Ethics granted us ethical approval. Informed consent was sought from all participants as well as from their parents when below the age of 16.

### 2.3. The Intervention—Cognitive Remediation Therapy

All CRT sessions were delivered face-to-face, and followed the structure and content outlined in a CRT manual specifically developed for children and adolescents with AN [34]. In accordance with previous CRT studies [14,35], no efforts were made to explore themes normally addressed in ED treatment such as food, weight or shape. One to two tasks were administered each session followed by discussion. The selection of tasks was based on the therapist’s clinical judgment, and chosen to address particular cognitive styles or difficulties specific to each individual. To help participants to engage in the meta-cognitive process, all tasks were accompanied or followed by questions encouraging the patients to reflect on their thinking processes. As an important part of CRT is to explore how experiences acquired in therapy can be transferred to real-life settings, homework tasks were introduced around session three or four. The aim of these tasks was for participants to use the knowledge acquired during CRT sessions, and to experiment with this knowledge outside the therapy context (e.g., in social settings, at school, or at work). Examples of these homework tasks can be found in the CRT Resource Pack [34,36].

### 2.4. Assessment—The CRT Treatment Evaluation Questionnaire

A treatment evaluation questionnaire was developed to investigate various aspects of treatment satisfaction and the participants’ perspective of change (see Table 2). The questionnaire combines CRT-specific items generated by clinicians and researchers at the unit where CRT was delivered, and items derived from treatment satisfaction questionnaires published by Kunnskapssenteret (the Knowledge Center for Health Services) [37] and RIKSÄT (a Swedish national register for ED treatment) [38]. The 20-item questionnaire was divided into four sections each exploring different aspects of treatment: (1) general attitudes towards CRT (items 1–5), (2) treatment-specific items (items 7–10), (3) the perception of change (items 6, 11–16c), and (4) the relationship to the CRT therapist (items 17–20). Aspects 1, 2 and 4 were labelled *treatment satisfaction* and aspect 3 was labelled *perceived change*. All but one item (item 13) were closed questions, and with one exception (item 9), response options were rated on a 5-point Likert scale. Response options differed depending on the wording of each item, i.e. where the response options to the question “*How do you feel about the number of sessions*” (item 8) were “Way too few”, “A little few”, “Just enough”, “A little too many” or “Way too many”, the corresponding response options to the question “How did you experience the collaboration between you and your CRT therapist?” were “Very poor”, Quite poor”, “Both”, “Quite good” or “Very good”.

### 2.5. Analyses

Results are presented using bar graphs (see Appendix A), with the response option being presented on the *x* axis, and number of responses on the *y* axis. As response options differed depending on the wording of each item, it was not possible to create bar graphs reporting collated results. Items were therefore divided into the following four categories: (1) General attitudes towards treatment (items 1–5), (2) Treatment-specific items (items 7–10), (3) The perception of change (items 6 and 11–16c) and (4) The relationship to the CRT therapist (items 17–20) and presented as individual item graphs. Item 13a-b was excluded due to the low response rate (N^13a^ = 5, N^13b^ = 9), and large variations in examples of areas that had become more or less difficult after the course of CRT.

## 3. Results

A detailed overview of the results from the CRT treatment evaluation questionnaire is presented in Appendix A. There were no voluntary drop-outs, and 19 out of 20 participants completed the entire course of treatment. Eighteen out of the 20 participants completed the treatment evaluation questionnaire post-CRT.

### 3.1. Treatment Satisfaction

In terms of the general assessment, 16 participants (89%) were either “very satisfied” or “satisfied” with the course of CRT they had received. Seventeen participants (94%) reported that the course of treatment had matched their expectations, and the same number of participants also reported having acquired skills that could be useful in their everyday life (with answers ranging from “very few” to “many”). All participants (N = 18) reported that the CRT sessions had been useful. As for treatment-specific items, 100% of the participants (N = 18) were either “very satisfied” or “extremely satisfied” with the tasks used during the sessions. Thirteen participants (72%) felt that the session length and the division between practical exercises and discussion were “just right”. Ten participants (56%) reported the total number of sessions to be “a little few”, whereas seven participants (39%) reported that the number of sessions were “just enough”. Overall, participants responded highly affirmative with regards to the therapist-specific items (items 17–20). For example, 15 (83%) participants reported that the collaboration between the patient and therapist was “very good”, and an equal number felt they had been treated with respect during the intervention and that the therapist had listened to them. Seventy-eight percent of the participants (N = 14) reported that the therapist had explained the tasks in a way that was easy to understand (item 20).

### 3.2. The Perception of Change

The perception of change was evaluated in relation to ED-specific items, but also in terms of other aspects of life, such as school or work, leisure time, friends and family. Eleven out of 18 participants (61%) reported that they were either slightly, or much more aware of their thinking style post CRT. Fifty-nine percent of the participants (N = 10) reported that ED-related distress was “more or less the same as before” CRT, and seven participants (41%) chose the same response when probed to appraise their relationship to their family post-CRT. Eight participants (47%) reported a “slightly better” relationship to their family after the intervention. As for items related to everyday functioning (item 15a-b), the majority of participants (61%, N = 11) reported that school/work was “more or less the same as before” CRT. Corresponding numbers for leisure time/friends was N = 8 (44%). Whereas 10 participants (56%) reported that the CRT sessions had had “a little” (N = 7), “quite a lot” (N = 2) or “a lot” (N = 1) of impact on their ability to change with regards to the ED, 44% of the patients (N = 8) reported that CRT had had either “very little” (N = 5) or “none not at all” (N = 3) impact on ED-related change. Results were almost identical with regards to perceived change in relation to school and/or work. The intervention appeared to have had limited impact on the participants’ perceived change with regards to leisure and/or social activities with six participants (40%) choosing the response option “very little”, five participants (33%) “little”, three participants (20%) quite a lot and one patient (7%) “a lot”.

## 4. Discussion

The aim of the current study was to investigate post-intervention feedback from a feasibility study on CRT for adolescents, and to delineate the individuals’ perspective in terms of general and specific attitudes towards treatment, and the relationship to the CRT therapist. Perceived change post-CRT was also investigated using ED-specific and non-ED-specific items. Overall, results indicate high levels of treatment satisfaction, both in terms of general attitudes towards treatment, treatment specifics (e.g. treatment length, content, tasks, etc.), and the relationship to the therapist, but somewhat limited perceptions of change.

### 4.1. Treatment Satisfaction

Tasks used in CRT are based on their potential playful capacity, and it is hypothesized that their simple, non-threatening nature help engage patients in treatment, and encourage curiosity rather than resistance [14,39,40]. This was supported in the current study where the majority of patients reported being either slightly or very satisfied with the tasks used to identify and challenge cognitive styles. Our results regarding task specifics are also in line with those recently published by Giombini et al. [20] supporting the use of board games and puzzles presented in the CRT Resource Pack [36]. Ten sessions is the length of treatment most commonly provided in both adult, child and adolescent CRT delivery. However, the results presented here call into question whether ten sessions are enough. More than half of the patients in our study reported that the number of sessions was “a little few”, and similar to the study by van Noort et al. [22], some of the patients actually requested additional sessions, an underreported phenomenon in patients with AN. In contrast to patient feedback in Wood et al.’s study from 2011 [40] where sessions exceeding 30 minutes were felt as being too long, the majority of patients in the current study reported the session length to be “just right”, and a few patients actually thought the sessions were a little short. In addition, the majority of patients in the current study communicated that they were satisfied with the treatment that they had received, and on an overall level, patients reported that the CRT sessions were both useful and relevant to their situation. Thus, in stark contrast to other treatment studies in EDs where adolescents with AN often report negative recollections of their treatment [41], CRT appears to provide a positive framework for this particular patient group.

High levels of dropout is a recurrent issue in treatment of AN, suggesting that a number of patients are either unwilling or unable to engage in treatment. Dropout rates for adolescents with AN who are hospitalized are reported as high as 24% [42], and even though dropout rates are less frequent in adolescents than in adults, a 95% completion rate, as reported in the current study, is unusual—and highly promising—for this group of patients. As treatment compliance has been shown to be a facilitator for recovery and a predictor of a successful outcome [43], it is paramount that we identify therapies that increase compliance and therapy adherence.

CRT for adolescents has been administered in a variety of modes including individual delivery [19,22,35], family-CRT [44], CRT delivered in collaboration with carers [45] and in groups [39,40]. In the current study, CRT was provided individually with one therapist delivering all treatment. One major advantage of individually delivered CRT is the possibility to tailor the treatment to each specific patient. In our case, CRT sessions were dedicated exclusively to the specific needs and circumstances of each individual, and it is not unlikely that such exclusivity could have had a positive effect in strengthening the therapist–patient bond. A good therapeutic alliance is known to predict treatment adherence and better outcomes in patients with EDs [46], especially young patients [47], and previous research in eating disorders suggests that treatment satisfaction is closely related to the way in which care is delivered [48]. In the current study, the vast majority of patients chose the most favorable response outcome (“very good”) when asked about the collaboration between the patient and the therapist, and the remaining therapist-specific items were answered equally, positively affirmative. Favourable outcomes, such as these, might reflect a number of aspects specific to CRT. Firstly, it is likely that the very nature of the intervention plays an important role in this type of feedback. In contrast to traditional ED treatment, CRT does not focus on core ED psychopathology such as weight, shape or eating. The lack of focus on ED symptomatology, which is often emotionally charged, might have rendered patients curious, rather than reluctant, and motivated rather than discouraged to engage and remain in treatment.

### 4.2. Perception of Change

In line with a recently published study by Giombini et al. [20], individuals in the current study experienced limited transferability of skills from the therapy context to their everyday lives. In Giombini et al.’s study [20], patients were asked to provide feedback on the CRT sessions as a way of evaluating the program. Although CRT was positively received by the majority of patients, a group of patients reported difficulties in understanding how CRT might be helpful in their everyday life, and what impact it would have on their recovery. In an adult CRT study by Easter and Tchanturia [49], similar findings are reported. This study used therapists’ feedback letter to investigate the implementation of CRT in the patients’ day-to-day life. Comparable to the current study, these patients also found it challenging when attempting to relate skills acquired in CRT to their everyday lives. A similar methodological approach, with comparable findings, was presented in a recently published study by Giombini et al. [50]. In their qualitative appraisal of young peoples’ experience of individual CRT, only 13% (N = 9) of the patients were able to describe how they could use knowledge acquired during CRT sessions in their daily life. In the current study, more than half of the patients reported that they had acquired either quite a few or many skills during CRT sessions that could be useful in their everyday life. However, when probed more specifically on whether CRT had impacted the ability to change—either with regards to the eating disorder or their everyday life—the majority of patients reported “Not at all”, “Very little” or “Little”. These results call into question the direct transferability of in-session learning, suggesting that more work needs to be done in order to help patients to channel new insights and behaviors from the inside to the outside of the therapy room.

To the authors’ knowledge, this is one of two [51] existing studies to explicitly investigate how individuals perceive everyday life and ED-related changes post CRT. Despite the majority of participants reporting being slightly more, or more, aware of how they reflect on their own thinking after CRT, our results indicate that they find the impact of CRT on the ability to change to be fairly limited. It could be argued that this is due to difficulties in evaluating one’s own change. In a previous study investigating self-reported executive functioning before and after CRT, Dahlgren and colleagues [3] showed that parents reported a greater extent of change in their child’s everyday behaviors, in comparison to the patients’ own reports. This could be due to biased post-intervention ratings based on anticipation or wishful thinking, or it could be because adolescents have difficulties in evaluating their own behavior and potential changes in behavior and attitudes. Another explanation is that changes in cognitions and behaviors require far more than ten CRT sessions to appear, or in our case, perceived by patients themselves.

## 5. Strengths and Limitations

Altogether, the participants in the current study appeared to find CRT useful and relevant, and the vast majority of patients reported being very satisfied with the relationship to the CRT therapist. However, since it is well documented that a good alliance between the patient and the therapist is central to engaging patients and motivating them to stay in treatment [46,47], it is possible that the role of the therapist played an important part in treatment adherence. For example, it is difficult to differentiate “treatment satisfaction” from “therapist satisfaction”. It is unknown whether the results would have changed if more than one therapist had delivered the CRT, and that such changes had been independent of the treatment per se. Further, we cannot exclude a therapist-specific contribution to the observed low dropout rate. Thus, it remains to be investigated if the results from the current study can be reproduced in other contexts with a number of therapists delivering the intervention remains. The study is also limited in terms of its sample size and the age range. It is likely that the perspective of change more easily can be reflected on and self-assessed by an individual at age 18 compared to someone at age 13. Further, as response options differed between items, it was not possible to report summary scores or merged data from the four different sections of the questionnaire. In-depth interviews could have served as an important adjunct to our questionnaire, allowing for qualitative analyses of the service user’s perspective.

Finally, the study is limited by not providing anonymous feedback forms. This is a limitation not just for the current study, but for all studies that have so far been published on patients’ perception of CRT. Patients’ reports are likely to be biased as all patients knew that the therapist would read their feedback forms. In addition, as AN patients often have a tendency of seeking social approval [52,53], this is especially important to take into consideration when evaluating patient feedback.

## 6. Conclusions

The current study provides one of the most detailed accounts of the patient’s perspective on CRT published for adolescent patients with AN. The findings highlight several important intervention aspects viewed through the eye of the receiver. On an overall level, patients reported CRT sessions as being both useful and relevant to their situation, and the majority of patients communicated that they were satisfied with the treatment they had received. On the other hand, and in line with a number of previous studies, the findings also indicate that patients perceived limited impact of CRT in terms of changes in their everyday-life functioning and their eating disorder. Such results warrant further investigation and a critical evaluation of treatment content, as well as of outcome measures used. Future studies might want to place an emphasis on crystalizing the primary purpose of CRT for adolescents with AN, and choose outcome measures appropriate to assess expected outcomes. If, as pointed out by Dahlgren and Rø [9], the main aim of CRT is to prepare the patients for subsequent treatment, future CRT studies should place further emphasis on motivational aspects of treatment and its likelihood of preventing drop-out, rather than focusing on the direct effect of CRT on measurable cognitive or behavioral changes.

## Figures and Tables

**Table 1:** Descriptive data for the sample (N = 20) at baseline.

	N	Mean (SD)	Range
Age (years)	20	15.9 (1.6)	13.1–18.7
BMI Percentile	20	10.2 (17.2)	0.0–28.0
Duration of illness ^1^	19	2.7 (2.1)	1–7
Global EDE-Q ^2^	20	3.4 (1.4)	0.6–5.4
BDI	20	32.2 (15.1)	7–58
STAI	20	58.6 (9.7)	43–78

*Note*. SD = Standard Deviation; BMI = Body Mass Index; ^1^ = Years, self-reported; ^2^ = Eating Disorder Examination Questionnaire Global Score [30]; BDI = Beck Depression Inventory [31]; STAI = State Trait Anxiety Inventory [32].

**Table 2:** The CRT Treatment Evaluation Questionnaire.

Item no.	Question
1.	Overall, how satisfied or dissatisfied are you with the course of CRT you have received? ^1^
2.	Before you initiated CRT, you were informed about the treatment. Was this information useful? ^1^
3.	How did your CRT experiences match the expectations you had prior to treatment initiation? ^1^
4.	To what extent did you feel that the CRT sessions were useful to you? ^1^
5.	Did you experience CRT as being relevant to your situation? ^1^
6.	Did you acquire any skills during CRT that might be useful in your everyday life? ^3^
7.	How do you feel about the length of the sessions? ^2^
8.	How do you feel about the number of sessions? ^2^
9.	How did you experience the division between practical exercises and discussion during the session (you can check as many boxes as you like)? ^2^
10.	What is your opinion on the tasks used during the sessions? ^2^
11.	How has the way you reflect on your own thinking changed during the course of CRT? ^3^
12.	To what extent do you experience distress in relation to your eating disorder now, compared to before you started the CRT treatment program? ^3^
13.	Are there other areas in your life which have become more or less difficult now, compared to before you started the CRT program? a) Example no. 1, b) Example no. 2
14.	What is your relationship to your family like now compared to before you started the CRT program? ^3^
15.	How are things working out in your everyday life now, compared to before you entered the CRT treatment program? a) School/work? b) Leisure time/friends? ^3^
16.	Do you think the CRT sessions have had an impact on your ability to change with regards to: a) Your eating disorder? b) School/work? c) Leisure time/friends? ^3^
17.	How did you experience the collaboration between you and your CRT therapist? ^4^
18.	Did you feel that you were treated with respect during the course of CRT? ^4^
19.	Did the CRT therapist listen to you? ^4^
20.	Did your therapist explain the tasks in a way that was easy to understand? ^4^

*Note*: ^1^ = Items assessing general aspects of treatment; ^2^ = Items assessing treatment-specific aspects; ^3^ = Items assessing the perception of change; ^4^ = items assessing the participants’ view of the CRT therapist. Items 1–5, 7–10 and 17–20 represent items specific *to treatment satisfaction*. Items 6, 11–15, and 16a,b represent items specific to *perceived change*. CRT = cognitive remediation therapy.

**Figure A1:**
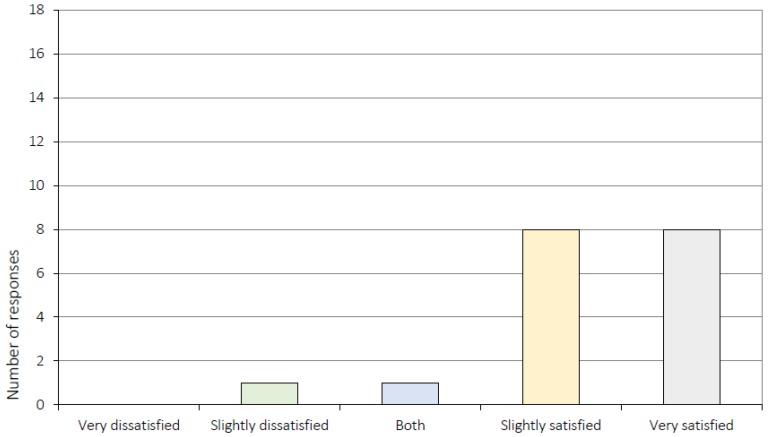
Overall, how satisfied or dissatisfied are you with the course of CRT that you have received?

**Figure A2:**
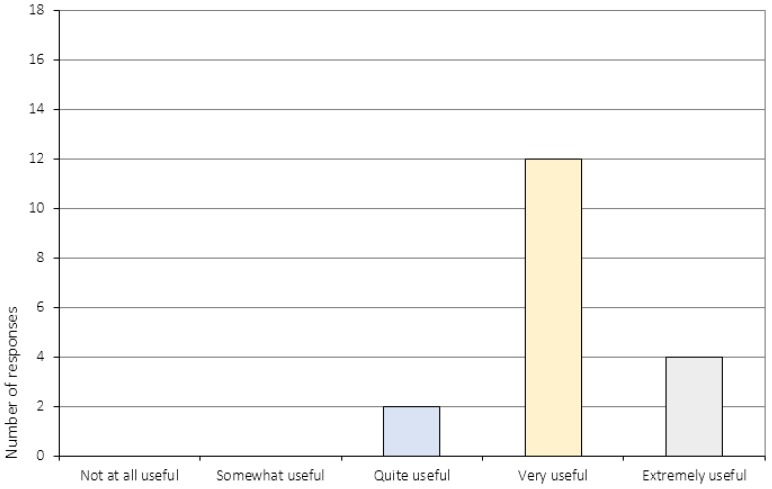
Before initiating CRT you were informed about the treatment. Was this information useful?

**Figure A3:**
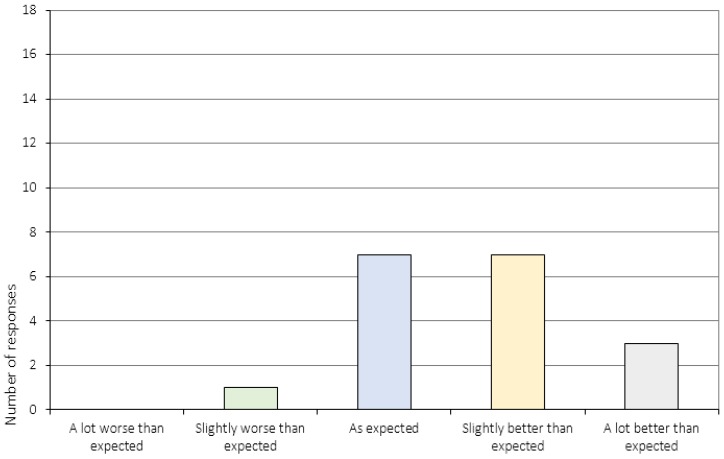
How did your CRT experience match the expectancies you had prior to treatment initiation?

**Figure A4:**
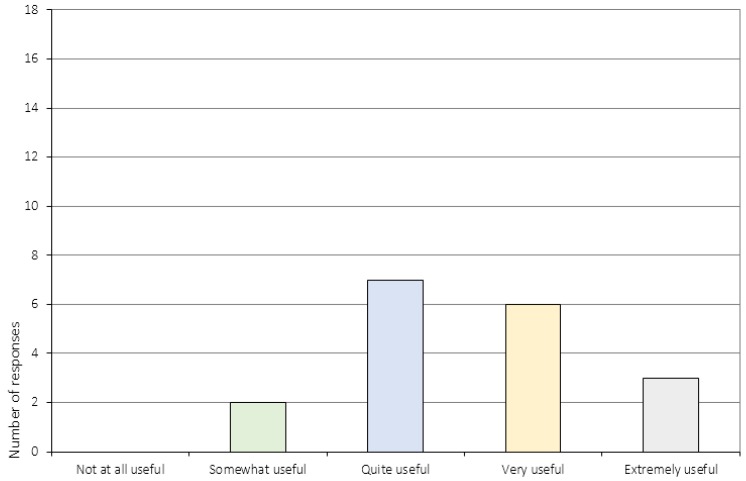
To what extent did you feel that the CRT sessions were useful to you?

**Figure A5:**
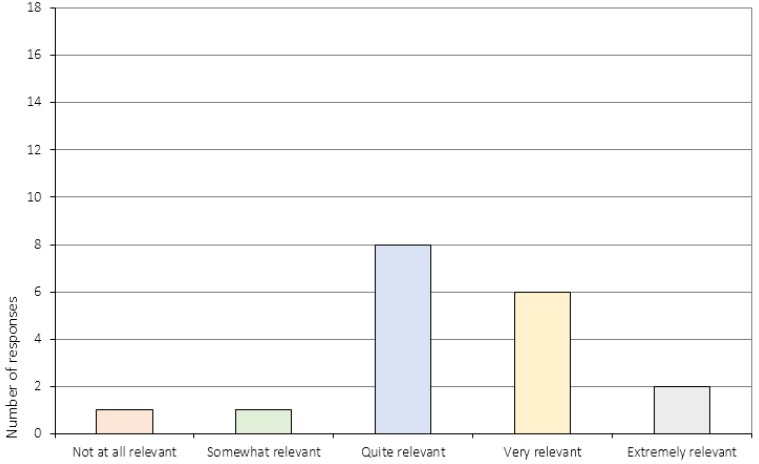
Did you experience CRT as being relevant to your situation?

**Figure A7:**
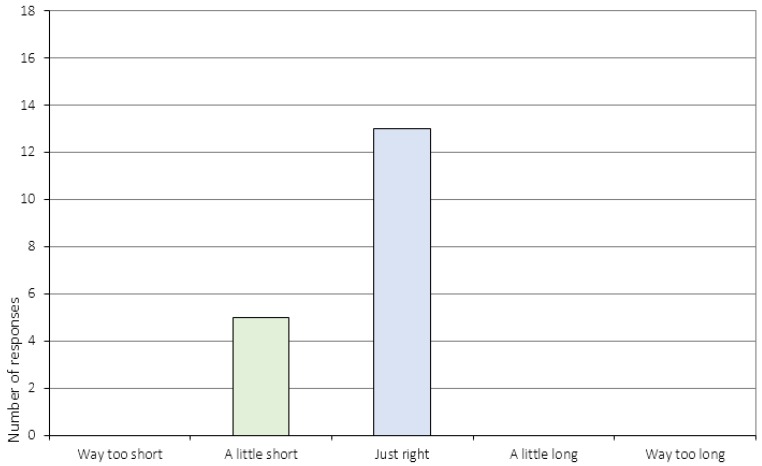
How did you feel about the the length of the sessions?

**Figure A8:**
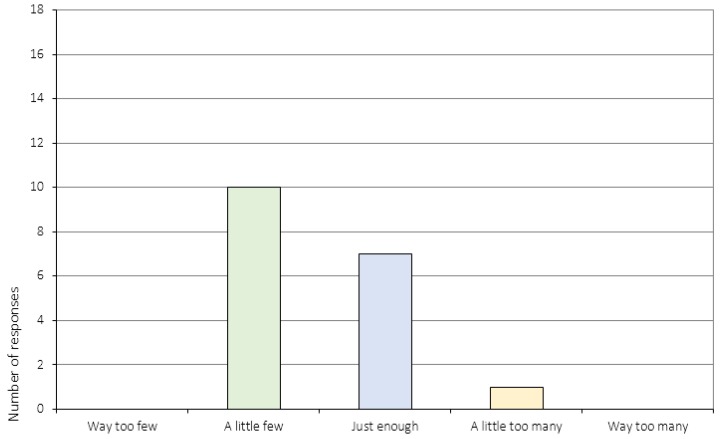
How did you feel about the number of session?

**Figure A9:**
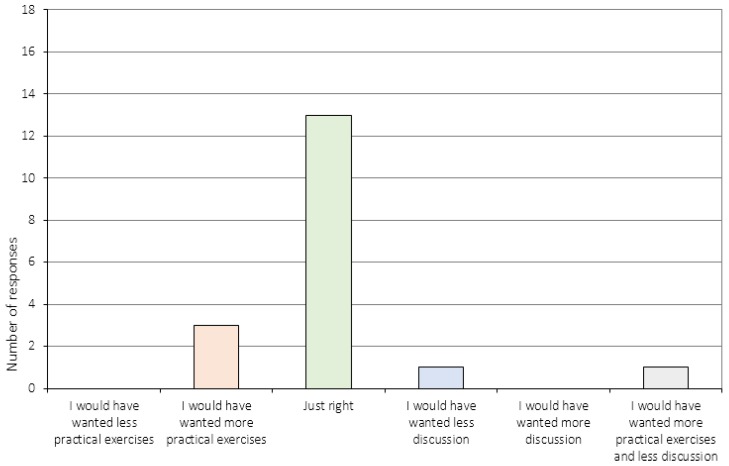
How did you experience the division between practical exercises and discussion during the sessions?

**Figure A10:**
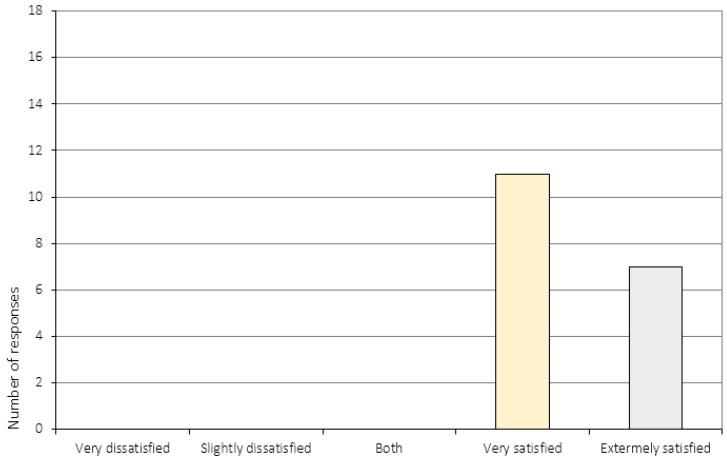
All in all, how satisfied or dissatisfied are you with the course of CRT that you have received?

**Figure A6:**
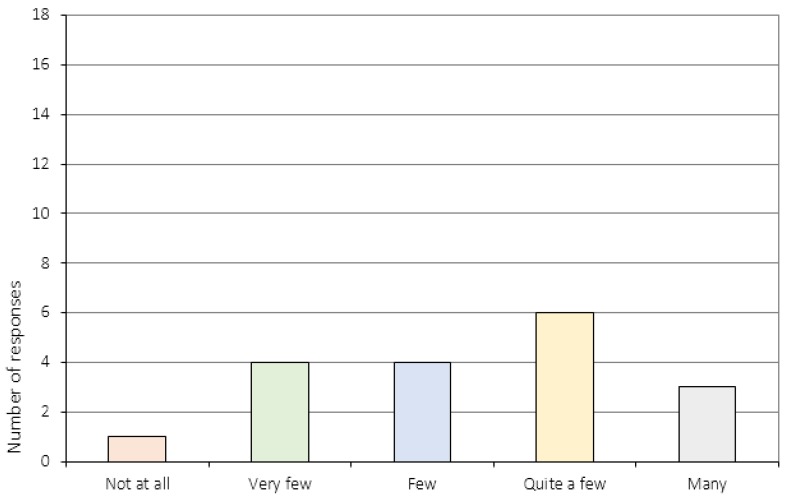
Did you acquire any skills during the CRT sessions that might be useful in your everyday life?

**Figure A11:**
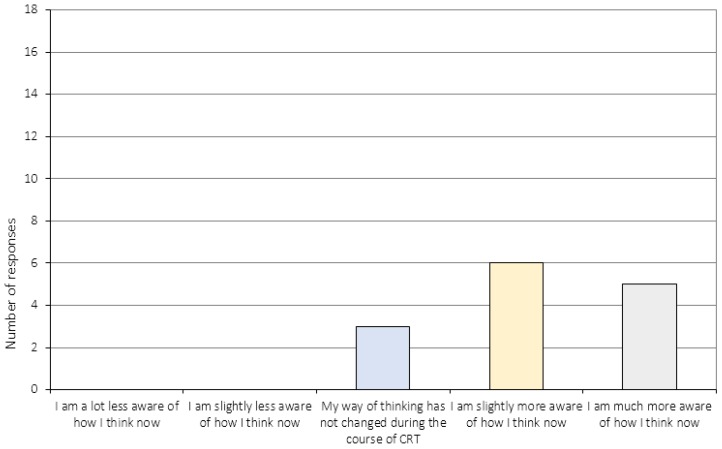
How has the way you reflect on your own thinking changed during the course of CRT? (*N = 14).

**Figure A12:**
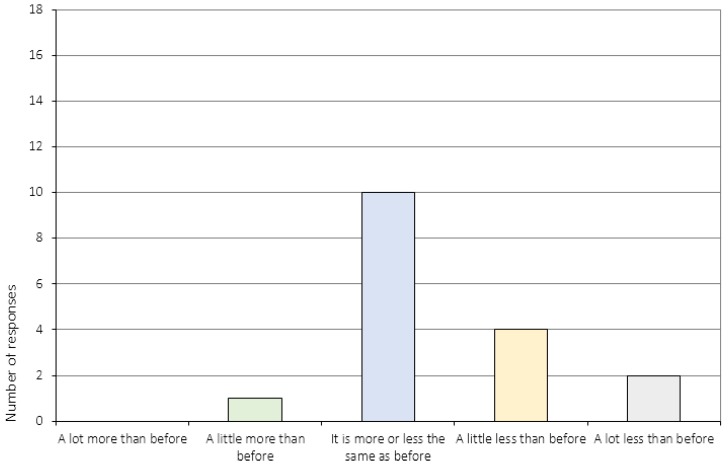
To what extent do you experience distress in relation to your eating disorder now, compared to before you started the CRT treatment program? (*N = 17).

**Figure A14:**
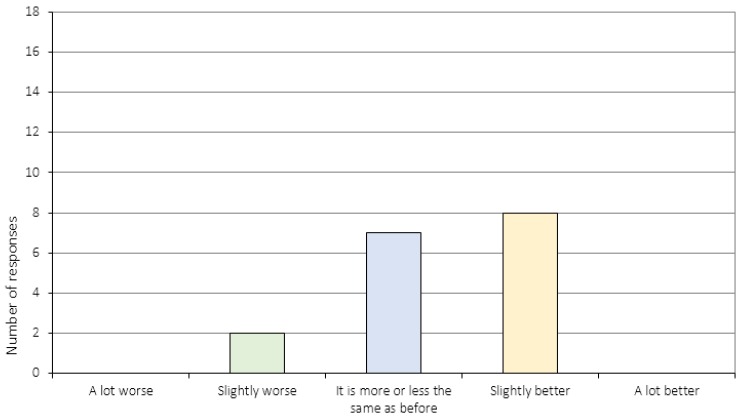
How is your relationship to your family now, compared to before you started the CRT treatment program? (*N = 17).

**Figure A15a:**
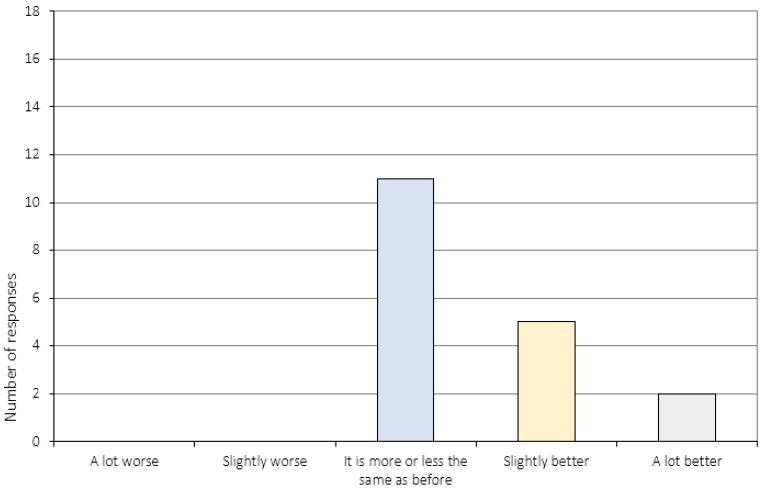
How are things working out in your daily life now, compared to before you started the CRT treatment program? (**a**) School/Work.

**Figure A15b:**
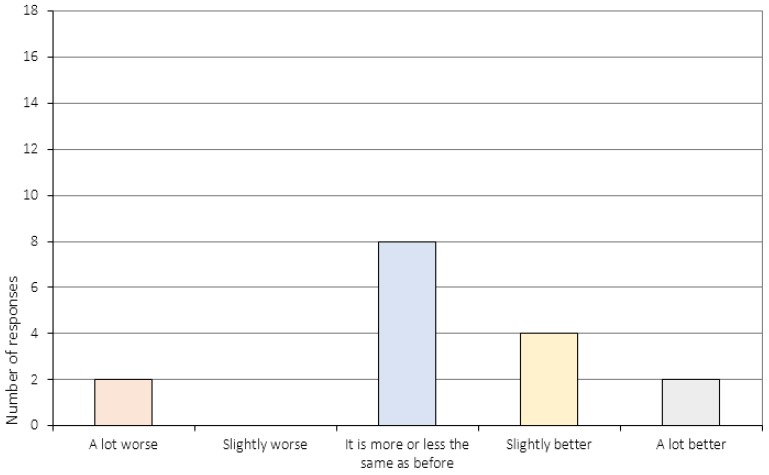
How are things working out in your daily life now, compared to before you started the CRT treatment program? (b)Leisure time/friends (*N = 16).

**Figure A16a:**
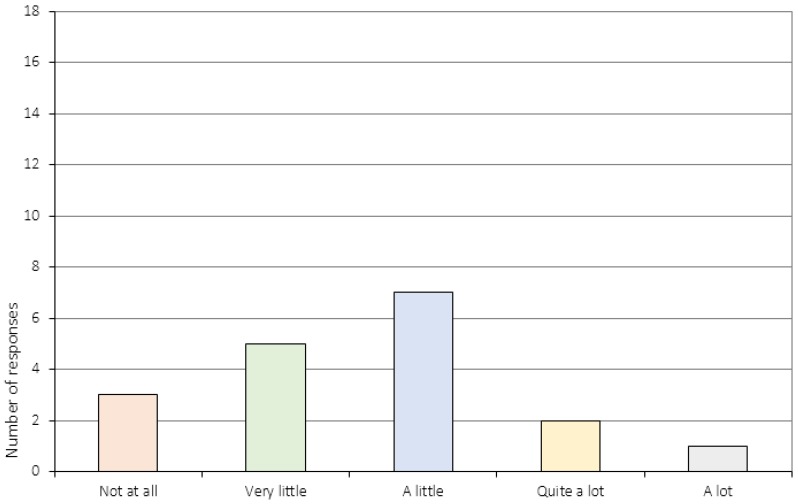
Do you think the CRT sessions have had an impact on your ability to change with regards to your eating disorder?

**Figure A16b:**
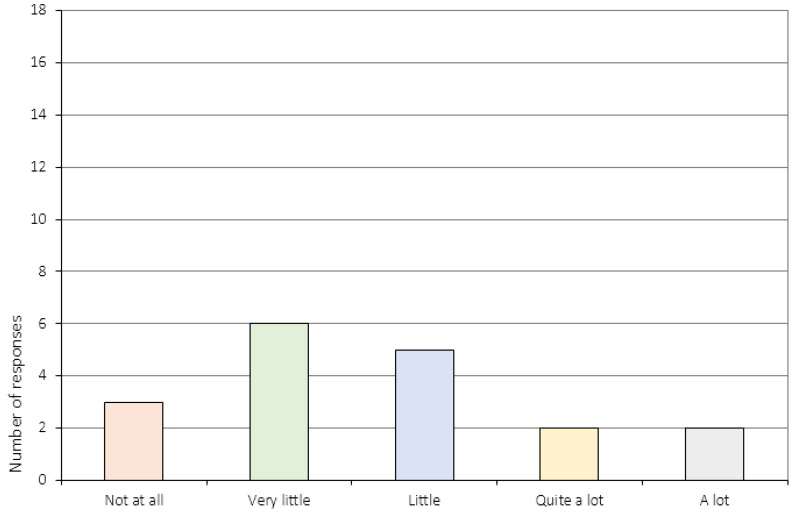
Do you think the CRT sessions have had an impact on your ability to change with regards to school/work?

**Figure A16c:**
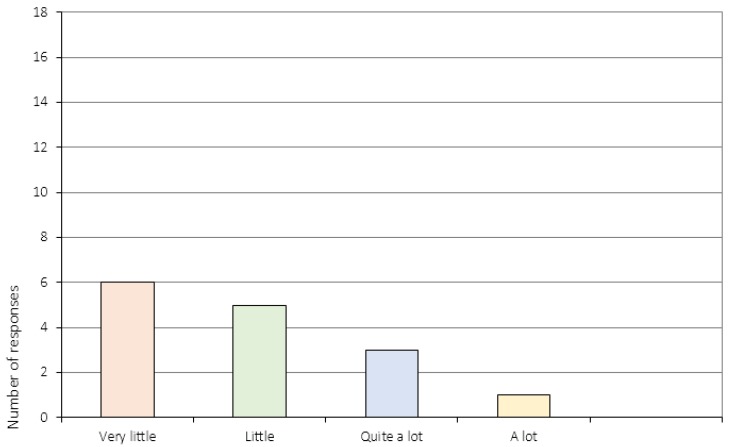
Do you think the CRT sessions have had an impact on your ability to change with regards to leisure time/friends? (*N = 15).

**Figure A17:**
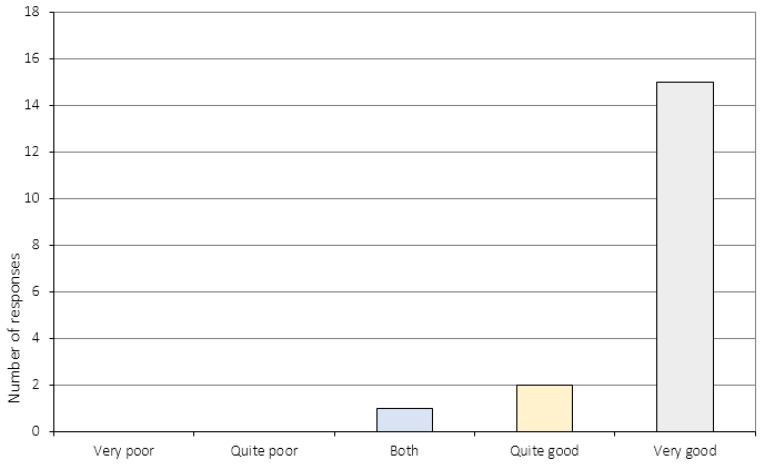
How did you experience the collaboration between you and your CRT therapist?

**Figure A18:**
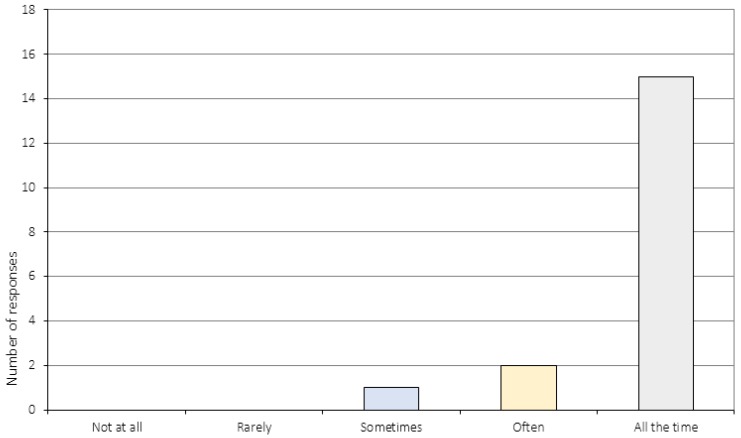
Were you treated with respect during the course of CRT?

**Figure A19:**
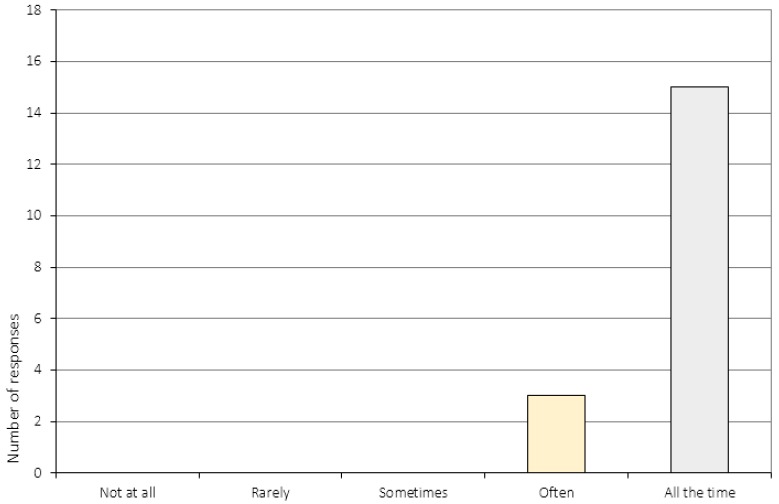
Did the CRT therapist listen to you?

**Figure A20:**
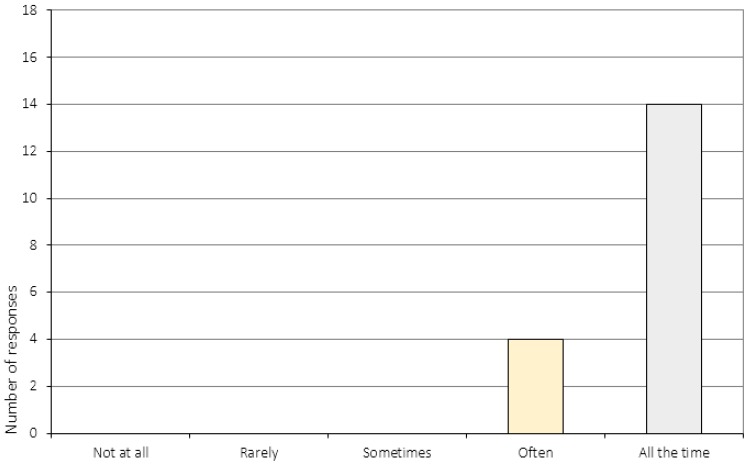
Did your therapist explain the tasks in a way that was easy to understand?

## Appendix A

Item-Specific Results from the Treatment Evaluation Questionnaire (N = 18). *Note.* Response options are presented on the *x* axis, and number of responses are presented on the *y* axis. Items with an asterisk (*) indicate fewer than N = 18 responses.
